# Failure Evaluation of Steel Plate Shear Walls in Multi-Storey Steel Buildings Under Seismic Excitation Using Convolutional Neural Networks

**DOI:** 10.3390/ma19050878

**Published:** 2026-02-26

**Authors:** Paolo Bonfini, Nikolaos Schetakis, Jurad Sukhnandan, Georgios A. Drosopoulos, Georgios E. Stavroulakis

**Affiliations:** 1 Alma Sistemi S.R.L., Via dei Nasturzi 4, 00012 Guidonia, Italy; 2 Quantum Innovation PC, Aristotelous 37, 73100 Chania, Greece; 3 Computational Mechanics and Optimization Laboratory (COMECO), School of Production Engineering and Management, Technical University of Crete, 73100 Chania, Greece; nischetakis@tuc.gr (N.S.); gestavroulakis@tuc.gr (G.E.S.); 4 Discipline of Civil Engineering, University of KwaZulu-Natal, Durban 4041, South Africa; juradnair@gmail.com; 5 Structural Engineering and Architecture Lab (SEAL), Department of Civil Engineering, International Hellenic University, 62124 Serres, Greece

**Keywords:** steel buildings, steel plate shear walls (SPSWs), computer vision, failure image prediction, convolutional neural networks (CNNs), non-linear time history analysis, seismic response

## Abstract

Multi-storey steel buildings may be susceptible to structural damage under seismic loading. Shear plate walls are often integrated in the structural system of this type of buildings in order to restrict the lateral response. This article aims, therefore, to propose a methodology for the automatic evaluation of failure on the shear plate walls of multi-storey steel buildings using computer vision. Physics-based non-linear dynamic finite element models have been developed and solved for a range of geometries, shear plate wall thicknesses and seismic loading from past events. Images depicting failure on shear plate walls given as equivalent plastic strain contour plots are included in the output data of the parametric simulations. Then, Convolutional Neural Networks (CNNs) are introduced, predicting the failure distribution on shear plate walls. The input parameters are the geometric properties of the buildings and the seismic event intensity, and the output parameters is the equivalent plastic strain images. This scheme was tested on random buildings with satisfactory accuracy. The proposed methodology can be adopted and used within structural digital twin solutions.

## 1. Introduction

A fundamental consideration in the seismic design of buildings is their dynamic response to ground-motion excitation. Design practice typically monitors global response quantities—such as inter-storey drift, base shear and fundamental period—to check serviceability and damage limits [1,2,3]. These response measures are estimated using simplified procedures incorporated in design codes, which cannot capture the ultimate structural response.

Alternatively, non-linear time-history analysis can be used to capture material and geometric non-linearity, local yielding and failure mechanisms at the expense of significant modelling effort and computational cost [4]. In common implicit integration schemes for dynamic time-history analysis, such as the constant average acceleration Newmark method, the stiffness is updated at each step and accuracy demands sufficiently small time increments, further increasing the computational burden for detailed structural models. In practice, this trade-off between fidelity and efficiency motivates complementary, data-driven surrogate models capable of predicting the non-linear structural response with lower computational cost.

A critical aspect of the seismic design of steel buildings is the choice of lateral load-resisting system during the design phase. Conventional solutions typically include diagonal cross bracing or moment-resisting frames. While effective, these systems can present challenges such as architectural constraints, limited energy dissipation capacity, or susceptibility to buckling [5]. In contrast, Steel Plate Shear Walls (SPSWs) have emerged as a highly effective alternative for providing lateral resistance [6]. A SPSW system consists of thin infill steel plates bounded by adjacent columns and beams, designed to yield and dissipate energy through the development of diagonal tension fields after buckling [7]. Compared to conventional bracing systems, SPSWs may offer higher stiffness, superior energy absorption, and stable hysteretic behaviour under cyclic loading. In addition, their use can lead to improved control of inter-storey drifts and more uniform distribution of seismic forces along the building height, thereby enhancing overall seismic resilience [7].

Steel plate shear walls (SPSWs) are widely adopted as lateral force-resisting systems for multi-storey steel buildings, providing high initial stiffness and stable hysteretic energy dissipation through tension field action after panel buckling [8]. Fundamental analytical and experimental studies established the mechanics of SPSWs and informed design approaches [9,10,11]. Design provisions (e.g., AISC 341-22) consolidate the best practices for steel shear plates to achieve ductile behaviour and reliable energy dissipation [3]. Yet, even for code-compliant SPSWs, accurately resolving local plastic demand (e.g., strain localization bands and their spatial distribution) under recorded earthquakes still relies on non-linear time history finite element analysis, especially when plate slenderness, boundary frame flexibility, and connection details are considered to determine system-level performance. This creates an appropriate framework for learning-based surrogate models that map earthquake demands and structural attributes directly to high-resolution fields of damage-relevant quantities, such as plastic strain, rather than only to global scalar responses.

Over the last decade, machine learning (ML) has gained attention across earthquake engineering for failure prediction, post-event decision support, and structural health monitoring (SHM) [12,13]. Reviews consistently report improved predictive capacity when ML augments or replaces parts of conventional workflows, while underscoring the need for physics-guided models and careful uncertainty treatment [14,15,16]. For structural response prediction under seismic loading, researchers have trained supervised learners (e.g., deep neural networks, random forests, kernel regressors) on simulation- or sensing-derived datasets to emulate the dynamic response and collapse capacity, often achieving much faster predictions compared with traditional finite element simulations [17,18,19].

Recent studies exploit deep learning, such as Convolutional Neural Networks (CNNs), Recurrent Neural Networks with Gated Recurrent Units (RNNs/GRUs), and hybrid Convolutional Neural Network–Long Short-Term Memory models (CNN-LSTM), to learn non-linear mappings from structural properties and ground-motion descriptors (or raw waveforms) to storey and time-history responses, achieving satisfactory accuracy and real-time prediction potential [20,21,22,23]. Physics-based deep learning further improves generalization with limited data and helps maintain the physical description of the problem [24]. Despite this progress, the dominant emphasis remains on global responses (drift, shear response, acceleration) and scalar performance metrics, noticing that comparatively fewer efforts target pixel-wise, full-field predictions of damage or plastic demand directly relevant to failure assessment.

Concurrently, computer vision has transformed how engineering fields handle spatially distributed quantities. Convolutional Neural Networks (CNNs) excel in dense, pixel-wise tasks (segmentation, super-resolution, image-to-image translation), with U-Net and its variants being adopted for learning high-fidelity mappings between images [25]. In mechanics, CNN-based surrogate models have been used to predict stress/strain fields from geometry and loading, producing full-field predictions competitive with finite element solutions at a fraction of the computational cost, and also enabling rapid parametric analysis and design space exploration [26]. In this context, relevant ML models can be used to learn the spatial distribution of plastic strains that governs damage localization and failure progression, information obtained from non-linear dynamic finite element simulations.

Among ML models, Convolutional Neural Networks (CNNs) are specifically designed to work with images: they hierarchically extract features, progressing from low-level local pixel patterns to high-level semantic representations [27].

Accurately capturing the structural response of multi-storey steel structures equipped with both lateral-resisting systems (cross-bracing and SPSWs) using conventional FEA can be computationally demanding and time consuming, particularly for extensive parametric investigations. Consequently, there is a clear need for an efficient alternative approach capable of providing rapid and reliable response predictions without the burden of repeated full numerical simulations. ML provides a solution to this problem by offering data-driven surrogate models capable of learning the complex non-linear relationships between structural parameters and seismic building response metrics. The potential of ML to serve as a fast and reliable predictive tool has been demonstrated in numerous engineering domains, yet its application to multi-storey steel structures remains relatively unexplored. The motivation for this research, therefore, stems from the need to bridge the gap between computational efficiency and accuracy in structural seismic predictions. By integrating finite element analysis with advanced machine learning techniques, this study aims to develop robust data-driven models that can accurately predict the structural seismic response. Achieving this integration will not only enable rapid seismic evaluation of multi-storey steel buildings, but will also contribute to the broader adoption of intelligent, performance-based design and digital twin frameworks in earthquake engineering.

For SPSW-equipped steel buildings, such full-field plasticity information is particularly valuable, since it may predict local strain concentrations at panel corners, along diagonal tension fields and near boundary element interfaces damage, also revealing the potential to repair the affected structural elements after severe seismic events. In addition, the capacity to predict the failure response of these elements without the need for advanced and computationally expensive non-linear dynamic finite element simulation offers more tools towards the structural health-monitoring for multi-storey steel buildings and their integration in structural digital twins.

Existing data-driven seismic studies seldom address SPSWs explicitly, and when they do, they typically focus on system-level indicators (e.g., base shear, storey drift) rather than pixel-wise plastic demand maps. To the authors’ best knowledge, there is a clear research gap at the intersection of (i) multi-hazard non-linear dynamic finite element-derived datasets using recorded ground motions on multi-storey SPSW frames and (ii) image-based deep learning models that predict the resulting plastic strain distributions and failure patterns with low computational cost. Bridging this gap could lead to rapid screening-level assessments of multi-storey steel buildings subject to seismic events, directly supporting performance-based design and detailing decisions.

The present study aims to cover this gap, proposing a computer vision-based data-driven framework that adopts non-linear time history finite element analysis to generate labelled image datasets of plastic strain fields for multi-storey steel frames with SPSWs under past seismic events. The plastic strain distributions derived from finite element analysis are treated as images and paired with input descriptors of geometric–structural configuration and ground motions. In our approach, we trained a CNN model that takes geometric and structural parameters as input and generates realistic plastic strain fields as output. In particular, CNN models were developed using U-Net-style encoder-decoder backbones augmented, as needed, with physics-motivated losses, to predict failure-relevant plastic strain maps for unseen structures and earthquakes. Compared to purely physics-based workflows, the learned surrogate model aims to provide (i) near-instantaneous plastic demand “images” suitable for screening and sensitivity analyses, (ii) the ability to generalize across topologies and plate parameters encountered in practical design, and (iii) compatibility with uncertainty quantification for model auditing and risk communication. By focusing on local field prediction rather than only on global metrics, the proposed approach aligns with emerging digital twin solutions in structural engineering, which combine high-fidelity simulation, measurement data, and ML to support rapid assessment and decision-making before and after earthquakes [16].

## 2. Dataset on Multi-Storey Steel Buildings with Shear Plate Walls

### 2.1. Introduction to the Concept of Parametric Simulations

This study adopts an image-based approach to propose a methodology for the fast and efficient prediction of the failure response of SPSWs used in multi-storey steel buildings to restrict the lateral response. A dataset of 3403 samples have been generated, providing a range of structural and loading parameters for multi-storey steel buildings with SPSWs. Non-linear dynamic finite element simulations have been performed using, as input parameters, the number of bays along the two horizontal directions, the number of storeys, the thickness of the shear plate walls, and the peak ground acceleration. Output parameters were images depicting failure on the shear plate walls in the form of equivalent plastic strain distribution. It is noted that other output parameters could be adopted to highlight the dynamic response of multi-storey steel buildings under seismic loading, such as the drift and base shear. However, the aim of this study is to predict the failure response of steel plate shear walls adopting computer vision, and, in this framework, equivalent plastic strain on shear plate walls is an appropriate parameter.

When zero equivalent plastic strain values appear, shear walls behave elastically and no failure on these elements arise. On the contrary, for non-zero plastic strain values, failure is initiated on shear walls.

For the selection of appropriate past seismic events that potentially trigger damage to the tested buildings, the following process was adopted. First, modal finite element analysis was performed for all parametric buildings, providing the values of the fundamental periods. Then, ground acceleration versus time past seismic events found in the Pacific Earthquake Engineering Research Centre (PEER) database were analysed and converted to response spectra diagrams using Seismosoft [28]. Finally, it was examined whether the fundamental periods of the numerically tested parametric buildings corresponded to the maximum values of the response spectra diagrams obtained from past seismic events. Using this process, a number of six past seismic events were chosen to provide the loading in the numerical finite element parametric simulations in the form of ground acceleration versus time diagrams along the x, y, and z directions. Using this loading, the overall duration of each seismic event was taken into account in the analysis. The chosen seismic events are shown in Table 1.

### 2.2. Finite Element Analysis Models for the Parametric Investigation

For the implementation of the dynamic simulations on multi-storey steel buildings using SPSWs, ANSYS (ANSYS 2021 R1) commercial finite element analysis software was adopted. A number of multi-storey steel buildings were tested using conventional steel cross-sections of the structural frames and a 250 mm thick concrete slab. In particular, the steel columns’ cross-section is 305 × 305 × 240-H, and the beams’ cross-section is 254 × 254 × 167-H section, according to the Southern African Steel Construction Handbook [29]. Beam-to-column joints were considered fixed in the finite element analysis model. A rectangular layout was adopted for the geometry of each building, using from three to ten bays per direction and three to ten storeys. The bay spanning (horizontal) dimensions were equal to 6 m × 4 m, and the inter-storey height was 3 m.

To provide a symmetric lateral stiffness to the buildings, one SPSW was placed in the middle bays of the external frames of every structure on all four facades. SPSW thicknesses equal to 10 mm, 15 mm, 20 mm, 25 mm, and 30 mm were considered. The SPSWs act as vertical cantilever plates, resisting lateral forces primarily through in-plane shear. A mechanism that often appears on shear plate walls subjected to in-plane cyclic shear loading is that the panel of the plate may develop local buckling in the compressive zone, which will then cause the development of post-buckling diagonal tension zones in the shear plate panel. Then, failure may occur due to tension, and this is a ductile failure mode that enables the system to dissipate significant seismic energy while maintaining structural stability [8].

**Figure 1:**
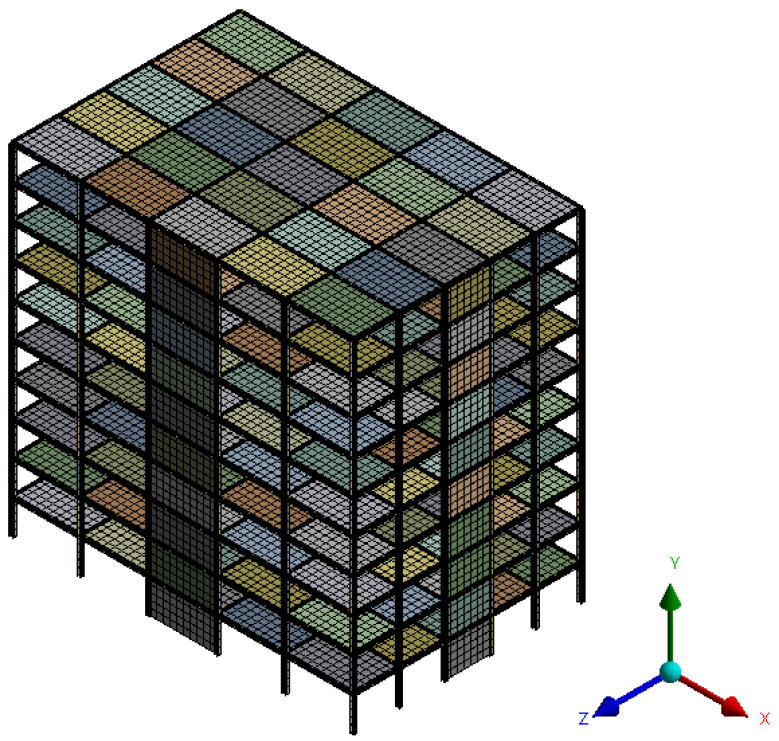
Finite element mesh for one of the buildings with SPSWs included in the dataset.

The finite element model of each multi-storey steel building consists of linear beam elements for the structural steel frames (beams and columns). For the shear plate walls and the concrete slabs, shell elements were used. Large displacement analysis was used, and a non-linear von Mises plasticity model was adopted to capture failure on the steel frames and on the shear plates, while the concrete slabs were assigned linear elastic properties. The chosen material properties are shown in Table 2. In Figure 1, the layout and finite element mesh for one of the multi-storey steel buildings is shown.

It is noted that, in the current study, SPSWs are integrated in three-dimensional multi-storey steel buildings to realistically describe the dynamic response of the overall system, as compared to existing studies that often consider SPSWs independently of the overall structural system. The dimensions of the SPSWs were set to fit the bay/panel dimensions, which are 6 m × 4 m, whilst the thickness varied parametrically. It is noticed that the SPSWs had a solid geometry, contrary to other studies where different geometric configurations, e.g., perforated SPSWs, were potentially considered.

In addition, the present effort extends the work presented in [19], where similar finite element models adopting bi-diagonal lateral bracing rods were adopted instead of SPSWs to resist the lateral actions.

**Figure 2:**
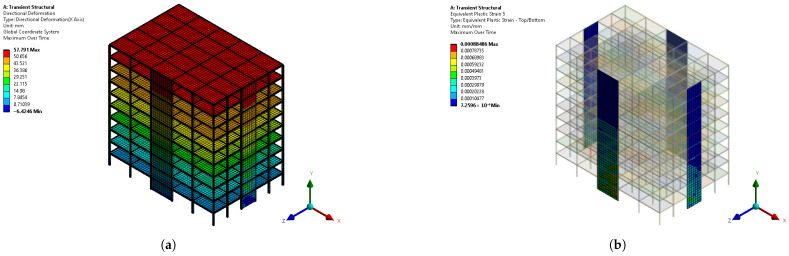
Example of multi-storey steel building’s response, as taken from the developed dataset: (**a**) Horizontal (along x) displacement distribution (mm) depicting also input geometry. (**b**) Accumulated output plastic strain distribution depicted on SPSWs.

As described in the following sections, the outputs of the proposed computer vision approach are the images depicting accumulated plastic strain distribution, and thus failure, on the steel plate shear walls. Figure 2a provides the horizontal displacement distribution (drift) of one building of the database and Figure 2b shows the accumulated plastic strain distribution on the steel plate shear walls for the same building. These images depicting plastic strain distribution on SPSWs are the outputs of the deep learning process.

## 3. Introduction to the Adopted Deep Learning Framework

This project focuses on simulating and predicting the effects of earthquake loading on synthetic buildings composed of bays arranged in a 3D grid of size R × C × K, where R is the number of floors and C and K are the number of bays along the building’s width and depth, respectively. Each building configuration is subjected to simulated seismic events, and the resulting response is analysed using finite element analysis (FEA).

This synthetic dataset is then used to train a CNN model that can predict post-earthquake plastic strain distributions developed on SPSWs, based on frame geometry and loading. By learning from the synthetic dataset generated using FEA simulations, the CNN aims to estimate the resulting plastic strain distribution on SPSWs across the building bays with high spatial resolution. This approach can potentially enable the real-time assessment of structural vulnerability without the need for computationally expensive simulations.

### 3.1. Data Preparation: Images

The accumulated image data consists of a collection of 3403 finite element simulations related to synthetic buildings rendered from four viewpoints, corresponding to the cardinal directions around the structure, i.e., front, back, left, and right view. For each building, we therefore have

- Four input images showing the structure from four cardinal directions;
- Four output images visualizing the maximum plastic strain distribution on SPSWs after an earthquake, also from the same four directions.

These views are captured both before and after the simulated earthquake loadings:

- The pre-earthquake images represent the building-frame geometry;
- The post-earthquake images display the contour plot of the plastic strain distribution computed via FEA.

**Figure 3:**
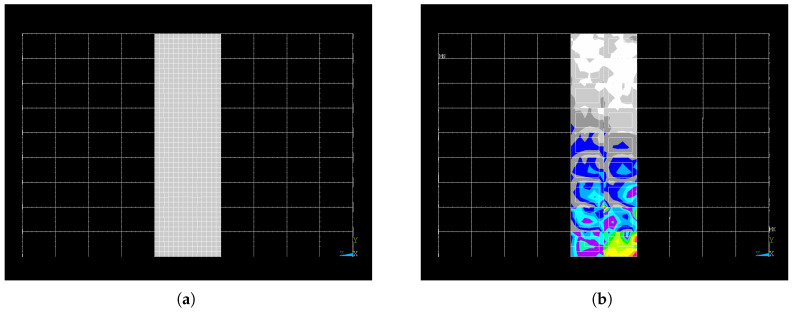
Example of paired dataset images: (**a**) The input frame geometry before the earthquake. (**b**) The corresponding simulated post-earthquake output plastic strain distribution on the SPSW.

The post-earthquake images use colour-coded bays to indicate the level of plastic strain: higher plastic strain regions are shown in warmer colours (e.g., red), and lower plastic strain regions in cooler tones (e.g., blue or green). Figure 3 presents an example of one building’s input and its corresponding output after the application of the earthquake loading, both from the front view.

As shown in the Figure 3, the structural analysis products are defined over a mesh of bays, where a bay represents a portion of a building bounded by structural support. For example, the building in Figure 3 is composed by a grid of nine bays along the rows (r) and ten bays along the columns (c).

It is noted that the conducted analysis focuses only on a subset of the total bays within each building (in the examples of Figure 3, the fourth and fifth column of bays). In this representation, the finite elements are shown as a mesh that subdivides the central bays. The mesh resolution and bay layout are consistent across all buildings, ensuring that plastic strain patterns can be compared between different designs. However, they may correspond to different physical sizes. This size and layout information is stored in accompanying metadata files provided alongside the images (see Section 3.2).

Each building image pair is therefore uniquely identified by

- The building’s ID;
- The view direction;
- The earthquake scenario;
- The shape of the bay grid.

**Figure 4:**
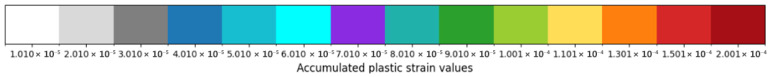
Colour mapping contour plot corresponding to plastic strain values given in Table 3.

For all remaining descriptions of the proposed deep learning process, the following accumulated plastic strain contour plot values correspond to the colours given in Table 3 and Figure 4. It is noted that the physical meaning of the low values of the plastic strain distribution is failure of low intensity.

### 3.2. Metadata

In addition to the images, each building–earthquake pair is associated with a set of metadata describing the structural and seismic parameters. These include the following:

- *Length, width, and height:* Measured in number of bays along the two horizontal directions (length and width) and height of each storey;
- *Wall thickness:* Structural thickness of each SPSW;
- *PGA (peak ground acceleration):* A measure of earthquake intensity;
- *POV (point of view):* The view direction from one of the four sides (A, B, C, or D);
- *Hz:* The fundamental frequency of each building (as obtained from modal analysis).

An example of these metadata is provided in Table 4.

### 3.3. Model Features

The metadata variables are used as predictors (*X*) in the CNN model, providing critical context about both the building’s geometry and the seismic input. They enable the CNN to learn how different structural configurations and earthquake characteristics affect the resulting plastic strain distribution.

While the metadata serve as the input features for the predictive model, the post-earthquake images—which show the plastic strain distribution—constitute the target variables (*y*). These images provide the ground truth output that the CNN is trained to predict.

In contrast, the pre-earthquake images were not used as inputs to the model, but were just employed for preprocessing purposes (see Section 3.4).

### 3.4. Data Preprocessing

Predicting the full plastic strain map of a SPSW as a single image is a highly complex task due to the high dimensionality of the output and the variability in structural layouts. To reduce this complexity and make the learning problem tractable, a “per-bay” prediction strategy was adopted. That is, rather than predicting the entire post-earthquake image at once, the model is was to predict the plastic strain at the level of individual bays. These local predictions can then be reassembled to reconstruct the complete plastic strain map.

Additionally, it is noted that the images—like the examples shown—often contain various artefacts that the proposed model is not expected to predict. These include the following:

- Ticks from the finite element mesh;
- Labels or annotations from the visualization tool;
- Slightly irregular or non-straight grid lines;
- Artefacts introduced by the finite element software (e.g., white boxes);
- Inconsistent bay sizes in pixel dimensions.

These elements are removed or reduced during preprocessing: the pipeline is specifically designed to filter out such noise and standardize the bay regions. This ensures that the model focuses solely on learning the meaningful plastic strain patterns and not on irrelevant visual distortions.

The result of this pipeline is a clean, well-aligned dataset of labelled bay-level image samples, which can be used to train a deep learning model. This strategy allows us to frame the problem as a structured, supervised learning task without the complexity of generating entire contour plot maps in one shot.

The predicting variables are assembled from the metadata introduced in Section 3.2, with two minor preprocessing steps. First, the POV feature is converted from a categorical variable to a numerical format using one-hot encoding (OHE), resulting in three new binary columns: POV_*A*_, POV_*B*_, and POV_*C*_. The category corresponding to the fourth and last point of view, POV_*D*_, is intentionally omitted to serve as the reference class and to avoid introducing artificial correlations in the data.

Secondly, since we are considering the images per-bay, we add two extra columns, r and c, indicating the row and column indices of each bay within the building grid, allowing for the model to learn spatial relationships between adjacent bays. The resulting metadata, which ultimately serve as input features to the machine learning model, appear as in Table 5.

### 3.5. Summary

After preprocessing and metadata integration, the dataset is organized into two components:

- *X*: The input feature matrix, including geometric and seismic metadata along with one-hot encoded orientation;
- *y*: The target data, consisting of images representing plastic strain contour plot distributions for individual bays.

Starting from 3403 simulations, each captured from four different viewpoints, we processed a total of approximately 14,000 images. From these, the pipeline extracted roughly 35,000 individual bay regions, which served as the model samples. Table 6 summarizes the shapes of the input *X* and targets *y*.

## 4. Deep Learning Model Design

The model is designed as a PyTorch convolutional neural network (CNN) capable of predicting the plastic strain experienced by each bay that belong to SPSWs of each the building following an earthquake. The model takes as input the building’s structural characteristics—such as its geometry and bay layout—along with the seismic input parameters, including the earthquake’s peak ground acceleration (PGA) and frequency.

### 4.1. Data Loading and Batching

The complete dataset consists of plastic strain image patches for individual bays on SPSWs, paired with a conditioning vector as described in Section 3.1. The conditioning vectors, representing the input *X* matrix, include the following (see Table 5):

- The building’s geometry (comprising *length*, *width*, *height*, and SPSW *thickness*);
- The seismic parameters (PGA);
- The viewpoint (encoded via one-hot vectors);
- The bay location indices (i.e., the row and column at which the bay lies within the bay grid).

The data is handled using a custom dataloader that

- Loads the conditioning vectors;
- Normalizes the numeric variables using a min–max scaler;
- Preserves the one-hot encoded POV variables;
- Loads the corresponding post-earthquake RGB image from disk;
- Resizes each image to 64 × 64 and normalizes it to the [0, 1] range.

The dataset is split into train/validation/test subsets using a standard 80/10/10 split, each further separated into batches of 256 samples.

### 4.2. Model Architecture

Our predictive model is effectively a convolutional decoder that generates the post-earthquake plastic strain (failure) image of a single building bay, conditioned on a vector of input parameters. In practice, it performs a regression from the conditioning vector to a full-resolution RGB image, using two main stages:

- A fully connected projection block transforms the input conditioning vector into a 2D feature map;
- A stack of transposed convolutional layers (a.k.a. “upsampling layers”) upsamples this feature map to the desired image resolution.

**Figure 5:**
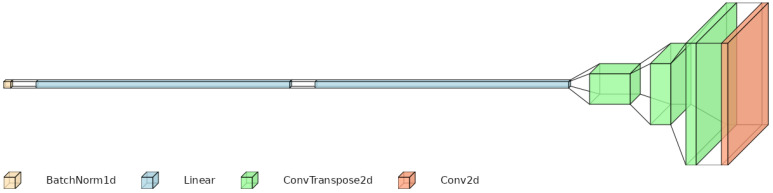
Model architecture. Diagram created using VisualTorch (https://github.com/willyfh/visualtorch accessed on 15 January 2026).

A schematic overview of the model architecture is presented in Figure 5. In particular, the conditioning vector is first normalized via a batch normalization (BatchNorm1), and then passed through two fully connected layers with ReLU activations. The output of these layers is reshaped into a low-resolution 2D feature map with a high number of channels. This feature map is progressively upsampled by a sequence of transposed convolutional layers (ConvTranspose2d), which increase the spatial resolution while reducing the number of channels. Finally, a convolutional layer (Conv2d) with Sigmoid activation maps the upsampled features to the desired three-channel RGB output, normalized to the desired [0, 1] range.

## 5. Results and Discussion

### 5.1. Training

**Figure 6:**
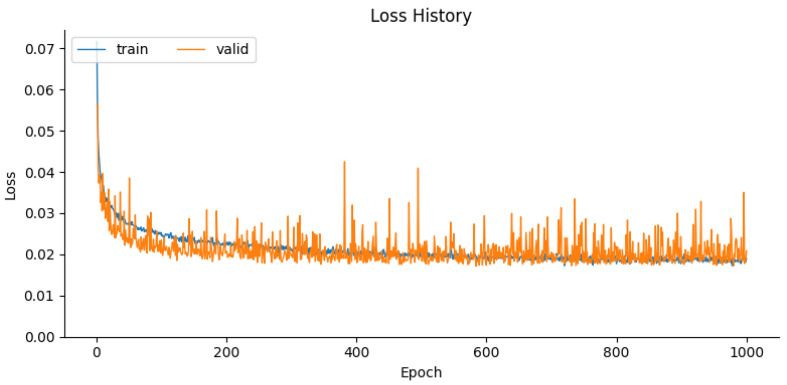
Training and validation loss over epochs, illustrating the model’s convergence.

The model is trained for 1000 epochs to minimize the difference between the predicted stress images and the ground truth post-earthquake images using a regression loss function, namely the Mean Squared Error (MSE). Early stopping based on validation loss is applied to retain the best model checkpoint. Training uses the Adam optimizer with a relatively low learning rate of 0.005 and a large batch size of 256. The loss curves for this procedure are shown in Figure 6.

**Figure 7:**
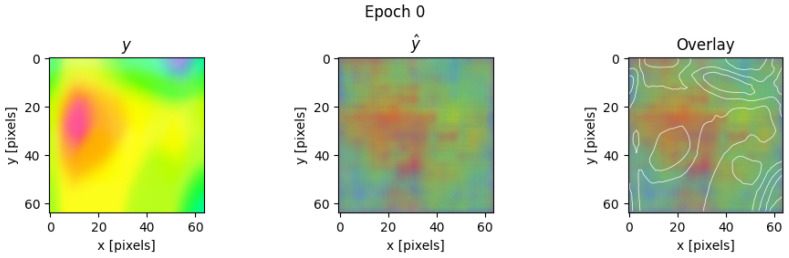
Example prediction from the validation set at epoch 0 showing the ground truth (**left**), model output (**centre**), and overlay comparison (**right**).

It is noted that training is performed on all bays extracted from all points of view. Because the viewpoint is included in the conditioning vector (POV), the model can directly learn to reproduce diverse plastic strain patterns specific to each perspective. As an example, Figure 7 provides a visualization of the model output for one individual bay at the end of the very first training iteration.

### 5.2. Predictions

The model achieves a Mean Squared Error (MSE) of approximately 0.017 on the test set, corresponding to a Root Mean Squared Error (RMSE) of about 13.0% relative to the normalized image intensity range [0, 1].

**Figure 8:**
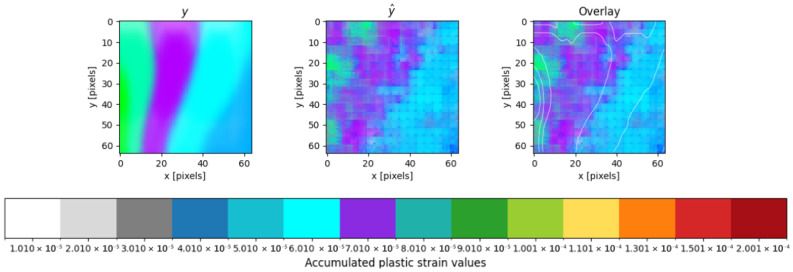
Example prediction for a single bay, before assembling, showing the ground truth (**left**), the model prediction (**centre**), and an overlay comparison (**right**).

An example of a CNN prediction on previously unseen (test) data is provided by the bay represented in Figure 8. The test sample is randomly selected from the total sample and follows the property distribution of the training set, as shown in Table 7.

It is observed that the predictions are slightly pixelated, which is a common artefact of the upsampling process in Convolutional Neural Networks. This is due to the model generating a low-resolution feature map that is then upsampled to the full resolution, which can introduce some quantization effects. This effect is partially mitigated during the reconstruction of the building from individual bays (Section 5.3).

By attempting a quantitative evaluation of the comparison between the ground truth and predicted images shown in Figure 8, it appears that zones of cyan-blue, cyan, and blue magenta, corresponding to plastic strain values between 5–7 $\times \;10^{-5}$, appear in both images, while some green-cyan and green in ground truth are not clearly visible in the predicted image.

### 5.3. Building Reconstruction

To visualize the predicted failure image for an entire SPSW, the model outputs for each individual bay are first reshaped to match the size and layout of the original bay template. This reshaping happens via bicubic interpolation, which partially smooths out the pixelated artifacts introduced during the upsampling process.

These predicted bay images are then systematically reassembled according to their spatial positions within the building’s grid, effectively reconstructing the full post-earthquake plastic strain map of the SPSW. Similarly, the ground truth bay images are reshaped and arranged in the same manner to reconstruct the ground truth building. This step is required due to minor variations in the sizes and aspect ratios of the original bays, which can lead to minor variations in the pixel dimensions of the reconstructed images. Reshaping all of them to a common template size allows for a direct visual comparison between predictions and ground truth at the building level.

**Figure 9:**
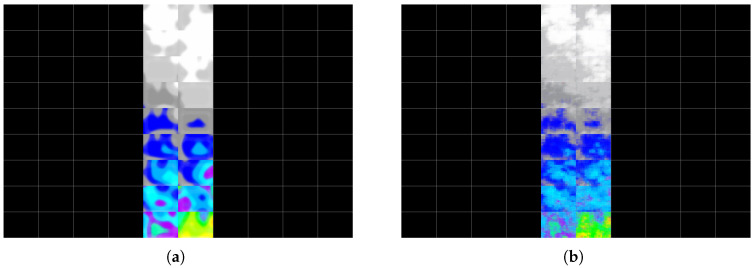
Reconstructed post-earthquake plastic strain distribution: (**a**) ground truth and (**b**) model prediction for the same building as in Figure 3.

Figure 9 shows the post-earthquake plastic strain distribution reconstructed at the building level, alongside the model prediction. The example refers to the same building shown in Figure 3.

### 5.4. More Testing Cases

**Figure 10:**
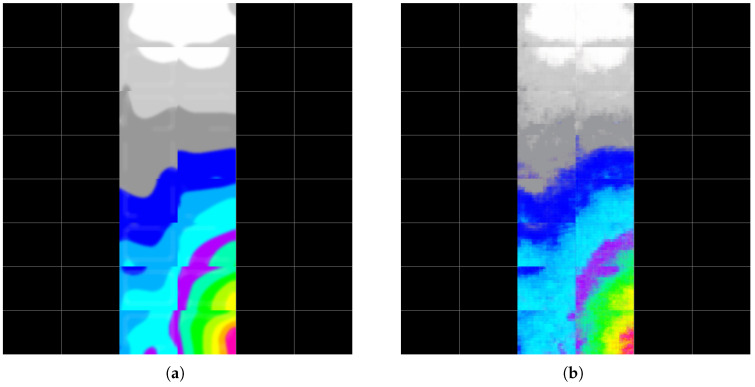
Reconstructed post-earthquake plastic strain distribution from the test dataset—Example 1: (**a**) ground truth and (**b**) model prediction.

**Figure 11:**
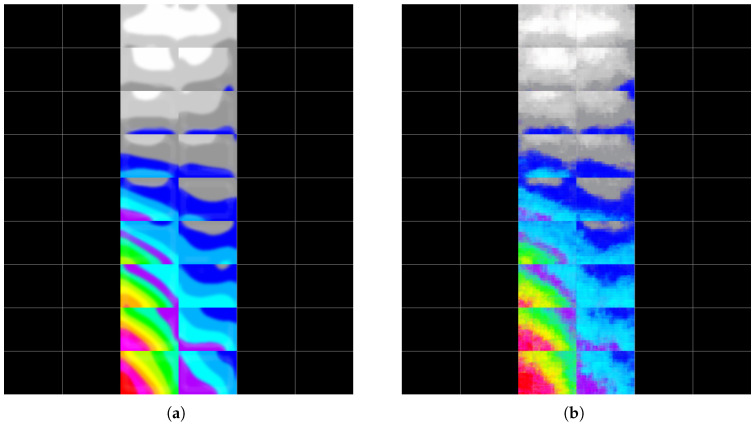
Reconstructed post-earthquake plastic strain distribution from the test dataset—Example 2: (**a**) ground truth and (**b**) model prediction.

To further highlight the capacity of the proposed deep learning approach to predict the failure response of SPSWs on multi-storey steel buildings, two more examples are provided using the testing dataset. For those cases, the prediction obtained from the adopted CNN approach is compared with the real image in the testing dataset (ground truth). In Figure 10 and Figure 11, two such examples are provided. For both cases, a Mean Square Error (MSE) of 0.002 was received. The input parameters adopted to generate failure on SPSWs for these examples are provided in Table 8.

In addition, authors have uploaded in https://doi.org/10.5281/zenodo.18336640 more input and output images, and made the material freely available for researchers.

Finally, in Appendix A, all the steps and the procedure adopted in the study to separate the individual bays of a building’s image are provided.

## 6. Conclusions

This article provides a methodology for evaluating the failure response of steel plate shear walls adopted to support multi-storey steel buildings against lateral, seismic actions using a deep learning approach. A dataset has been developed using non-linear dynamic finite element analysis on a range of multi-storey steel buildings. Input parameters included undeformed steel frame data providing geometry characteristics such as bay and storey numbers, as well as the thicknesses of steel plate shear walls, the fundamental frequencies of each building, and peak ground accelerations of the seismic loading events. Output parameters were images providing failure on steel shear walls in the form of accumulated plastic distribution.

To capture the plastic strain distribution, a Convolutional Neural Network model was developed and trained using the mentioned input–output parameters. The results indicate that the comparison of the predicted plastic strain distribution on shear plates is close to the one provided in the dataset. Mean Squared Error values between 0.002 and 0.017 were obtained in this study.

The proposed methodology can be adopted to provide a fast prediction of the ultimate response of multi-storey steel buildings under seismic actions. This approach can assist structural design tasks when evaluation of the response of a large number of buildings is needed and where the manual and computationally expensive simulation of every building is challenging.

The adopted metadata used as input on this study is relatively coarse. This may limit the model’s applicability. Therefore, this study can be enhanced and the model’s applicability can be improved by developing more dataset points that describe a greater range of building patterns and generate more dense inputs for the metadata. Experimental validation, even in smaller-sized buildings adopting some scaling, would also increase the impact of the proposed approach.

It can also be extended, by incorporating the image recognition part proposed here, in more holistic digital twin solutions. Thus, this work can be considered as a step towards developing more complete digital twin solutions where a real-time monitoring of the structural system is adopted and relevant information with the digital counterpart of the monitored building is exchanged. Within this framework, accelerations of the investigated building will be measured, providing seismic response data. This can be considered by the digital twin platform, in addition to the geometric characteristics of the building, towards predicting the failure response of SPSWs using the computer vision ML approach shown in this study. The response of the overall structural system and the influence of SPSWs on the dynamic response can be further evaluated in future work.

## Figures and Tables

**Table 1:** Seismic events chosen for the non-linear dynamic finite element simulations [19].

Name	Magnitude	Epicentre Distance (km)	Site Class	Peak Ground Acceleration
Irpinia, Italy-01	6.9	30.35	B	0.29 g
Northridge-01-California	6.69	45.77	B	0.168 g
Northridge-01-California	6.69	25.42	B	0.2458 g
Parkfield	6.19	17.64	C	0.38 g
Helena Montana-01	6.0	2.07	C	0.146 g
Imperial-Valley-01	6.53	8.54	D	0.31 g

**Table 2:** Material properties for structural steel and concrete [19].

Material	Law	Young’s Modulus	Poisson Ratio	Yield Stress	Ultimate Stress
Structural Steel (S355JR Grade steel)	Bilinear Isotropic hardening	200 GPa	0.3	355 MPa	365 MPa
Concrete	Isotropic Elastic	30 GPa	0.18	–	–

**Table 3:** Colour mapping for equivalent plastic strain values.

Colour	Accumulated Plastic Strain Values
White	$1.000\times 10^{-5}$
Light Gray	$2.010\times 10^{-5}$
Dark Gray	$3.010\times 10^{-5}$
Blue	$4.010\times 10^{-5}$
Cyan Blue	$5.010\times 10^{-5}$
Cyan	$6.010\times 10^{-5}$
Blue Magenta	$7.010\times 10^{-5}$
Green Cyan	$8.010\times 10^{-5}$
Green	$9.010\times 10^{-5}$
Yellow Green	$1.001\times 10^{-4}$
Yellow	$1.101\times 10^{-4}$
Orange	$1.201\times 10^{-4}$
Magenta Red	$1.301\times 10^{-4}$
Magenta	$1.401\times 10^{-4}$
Red	$1.501\times 10^{-4}$

**Table 4:** Metadata of the dataset (restricted view showing only the first few bay examples).

ID	Length	Width	Height (m)	SPSW Thickness (mm)	PGA (g)	POV	Hz
0001	3	4	3	10	0.2458	B	5.09
0002	4	5	3	10	0.2458	D	4.30
0003	5	6	3	10	0.2458	A	5.40
0004	7	8	3	10	0.2458	C	4.30
0005	8	9	3	10	0.2458	B	3.94

**Table 5:** Extended metadata, including bay-level information (restricted view showing only the first few bay examples).

Image	Length	Width	Height (m)	SPSW Thickness (mm)	r	c	PGA (g)	POV_*A*_	POV_*B*_	POV_*C*_	Hz
building_1_A_bay_1	3	4	3	10	1	2	0.2458	1	0	0	5.09
building_1_A_bay_2	3	4	3	10	0	2	0.2458	1	0	0	5.09
building_1_D_bay_1	3	4	3	10	2	2	0.2458	0	0	0	5.09
building_5_A_bay_1	4	5	3	10	1	2	0.2458	1	0	0	4.30
building_5_B_bay_1	4	5	3	10	0	2	0.2458	0	1	0	4.30

**Table 6:** Overview of the dataset structure.

Name	Shape	Data Type
X	(∼35,000, 11)	Metadata vector
y	(∼35,000, h, w, 3)	RGB image (target)

**Table 7:** Min and max values of the train and test dataset ^1^.

	Length	Width	Height	Thickness	PGA	Hz
min	2	3	3	10	0.146	0.920341
max	9	10	10	30	0.380	8.390196

^1^ Where length, width, and height are the building sizes expressed in number of bays, PGA is the peak ground acceleration, and Hz is the fundamental frequency of building considered.

**Table 8:** Input parameters for example predictions.

Example	Length	Width	Height	SPSW Thickness	R	C	PGA	Fund. Freq. (Hz)	POV_A	POV_B	POV_C
Example 1 (Figure 10)	5	6	8	25	7	3	0.1680	2.0087	1	0	0
Example 2 (Figure 11)	5	6	9	30	4	2	0.2458	1.7626	1	0	0

**Figure A1:**
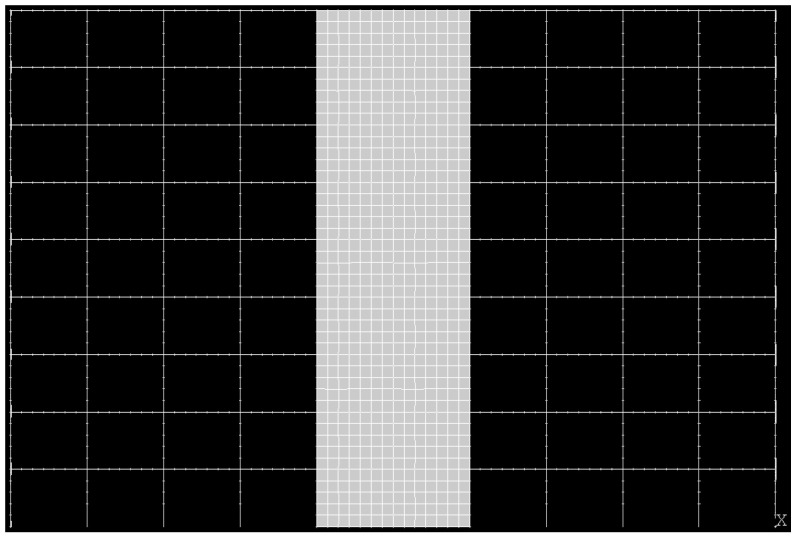
Filter out non-structural pixels and crop to the relevant building region.

**Figure A2:**
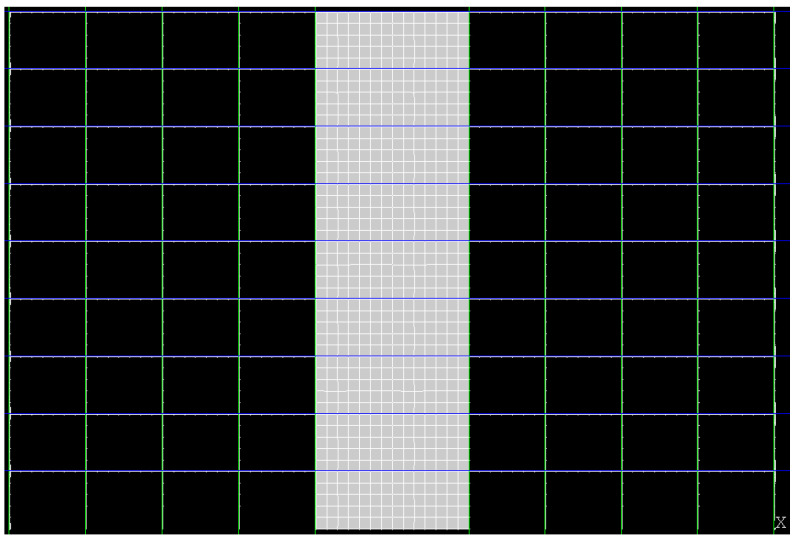
Detect bay grid layout using edge detection.

**Figure A3:**
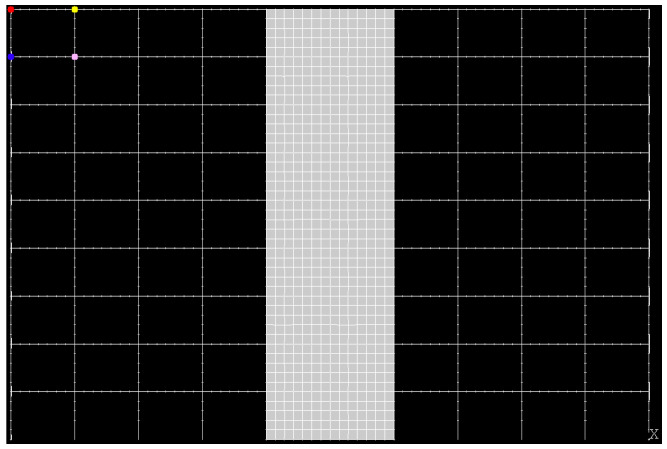
Identify the upper-left intersection points between the detected grid edges.

**Figure A4:**
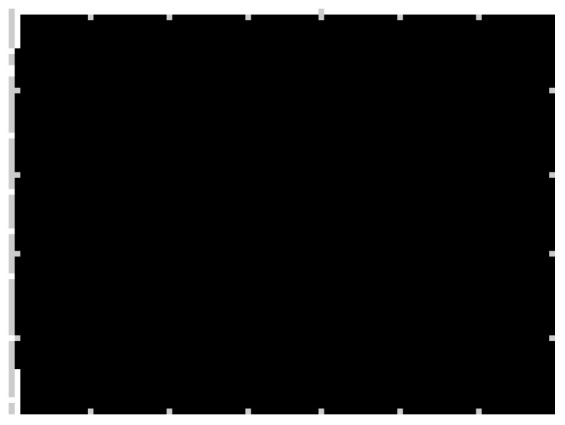
Extract a template bay region from the top-left corner of the grid.

**Figure A5:**
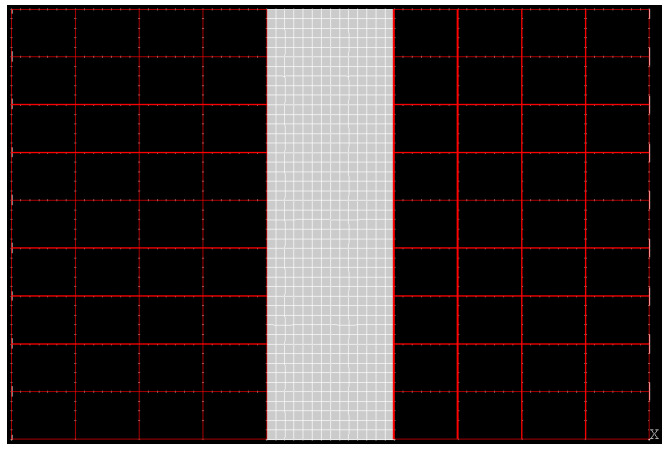
Detect all bay regions by matching the template across the image.

**Figure A6:**
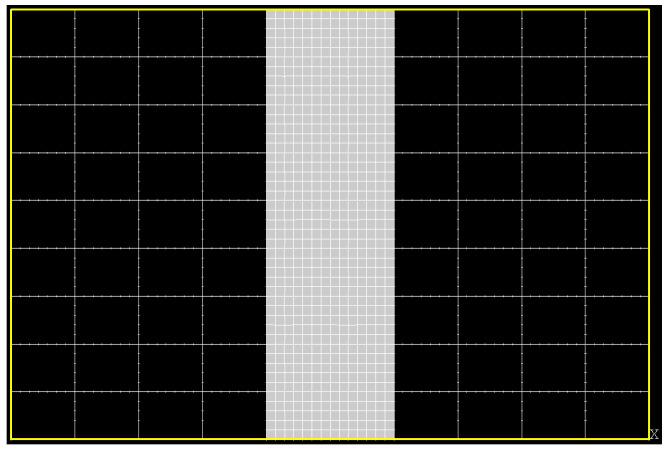
Draw a bounding box.

**Figure A7:**
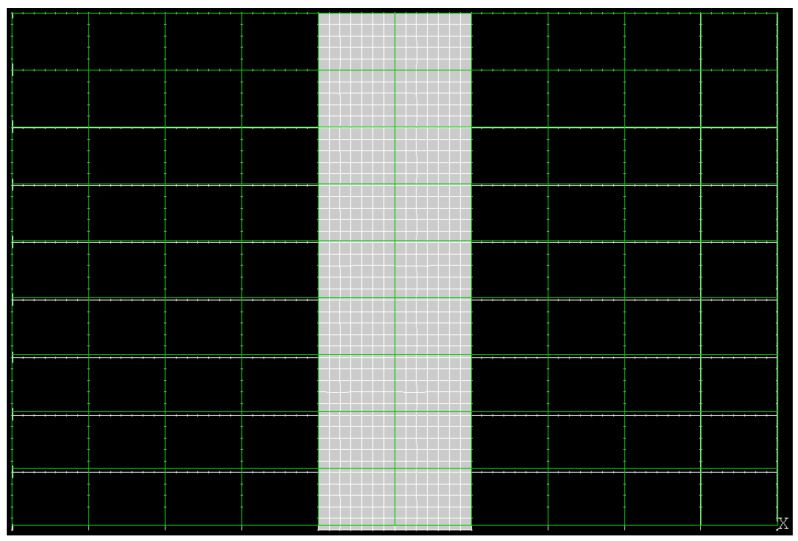
Draw a uniform grid.

**Figure A8:**
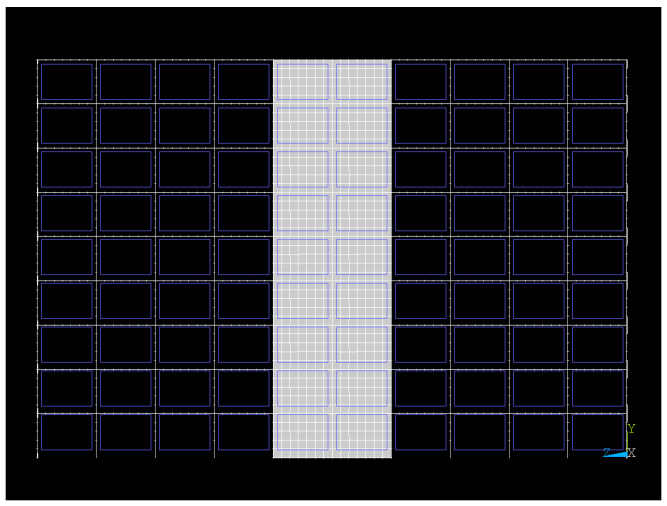
Shrink all the cells.

**Figure A9:**
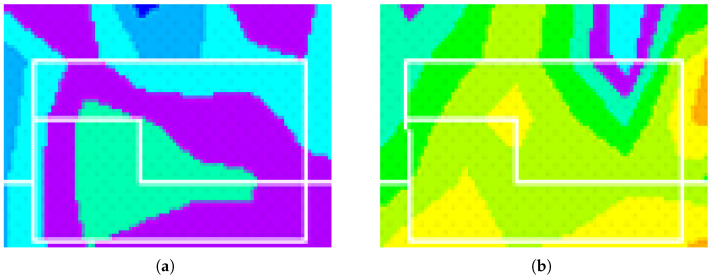
Extraction of bottom bays of the building shown in the right panel of Figure 3: (**a**) bottom-left bay and (**b**) bottom-right bay.

**Figure A10:**
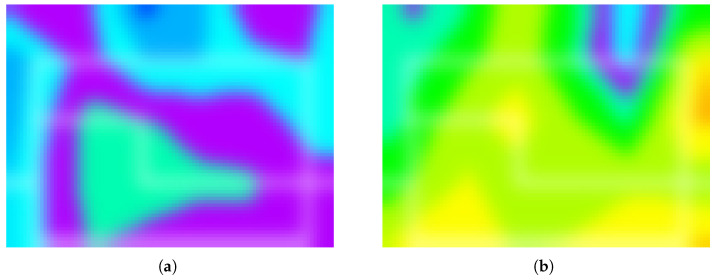
Smoothed bay extractions corresponding to the lower part of the grid: (**a**) bottom-left bay and (**b**) bottom-right bay.

**Figure A11:**
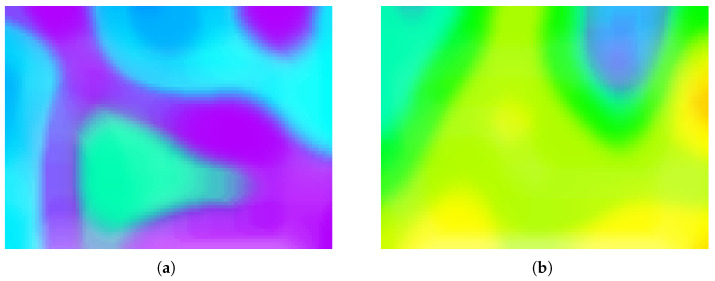
Filtered bay extractions corresponding to the lower part of the grid: (**a**) bottom-left bay and (**b**) bottom-right bay.

## Data Availability

Data related to the study and input–output images used to train the proposed CNN models can be found in https://doi.org/10.5281/zenodo.18336640.

## Appendix A

In this section, we outline the procedure used to separate the individual bays of a building’s image; this procedure is essential to the preprocessing described in Section 3.1.

This step is conducted using a Computer Vision (CV) pipeline that automatically identifies the bays on the simpler pre-earthquake images and then applies the same segmentation to the post-earthquake images.

The process begins with each raw input image, where we first isolate the structural content by filtering out background pixels and cropping to the bounding region of the building (Figure A1). This ensures that the analysis focuses exclusively on the meaningful geometry. Next, we detect the underlying bay grid by identifying the most prominent vertical and horizontal edges (Figure A2).

The following steps involve extracting a template from the top-left cell of the grid, which serves as a reference for the bay structure. First, we identify the upper-left intersection points of the detected grid edges (Figure A3). Next, a template is extracted from the top-left bay using the previously identified intersection points (Figure A4).

Template matching is then used to locate all other bay regions that resemble the extracted template (Figure A5). From the matched grid, we compute a bounding box that encloses the full bay layout (Figure A6). We then draw a structured grid of rectangles, each with dimensions equal to those of the template (Figure A7). This ensures a consistent segmentation into cells of equal size. However, the cell grid is only an approximation of the actual bay layout, since the bays may not be perfectly aligned or may vary slightly in size. To capture the actual content of the bays and avoid the grid lines and ticks, we first slightly shrink the grid cells (Figure A8).

This grid defines the effective segmentation of the image into individual proxy-bay regions, which we can then apply to the corresponding post-earthquake images. While this process does result in minor loss of edge information around each bay, it allows us to focus on the core structural content and avoid artefacts such as grid lines and mesh ticks.

After extraction, each bay image is resized to match the original template shape via bicubic interpolation. While this does not preserve the exact size of every bay in the image—since some may vary by a few pixels—it provides a consistent target size across all samples. For our purposes, this caveat is acceptable as it ensures uniformity in the training dataset.

For the post-earthquake image example shown in the right panel of Figure 3, the cropped bays of interest extracted from the bottom row appear as in Figure A9. To reduce high-frequency noise such as the grey pixels and the artefact segments visible in the images of Figure A9, a Gaussian blur is subsequently applied (Figure A10). This is ultimately followed by a median filtering operation, which is specifically applied to enhance the clarity of the extracted features by reducing residual noise while preserving important structural details (Figure A11). Finally, the dark bays—not used in the analysis (such as the first and last 4 columns in the pre-earthquake image of Figure 3)—are excluded from the dataset.
